# Patterns of Relapse in Squamous Cell Carcinoma of the Tonsil - Unilateral vs. Bilateral Radiation in the HPV-Era

**DOI:** 10.7759/cureus.322

**Published:** 2015-09-10

**Authors:** Allison Ye, Katherine L Bradley, Hosam Kader, John Wu, John H Hay

**Affiliations:** 1 Department of Radiation Oncology, British Columbia Cancer Agency - Vancouver Cancer Centre; 2 Department of Oncology, Queen Alexandra Hospital

**Keywords:** tonsil cancer, squamous cell carcinoma, head and neck, radiation, unilateral, bilateral, hpv, recurrence, p16, human papillomavirus

## Abstract

Objectives: In the pre-human papillomavirus (HPV) era, unilateral radiation therapy (URT) for tonsil cancer was associated with low contralateral failure rates and had less toxicity than bilateral radiation therapy (BRT). This study explores the validity of URT in HPV-positive tonsil cancers.

Methods: Tonsil squamous cell carcinomas (SCC) treated (typically with 70 Gy radiation and Cisplatin-based chemotherapy) between 2001 and 2007 were reviewed. Retrospective p16 immunohistochemistry staining was undertaken. Baseline, treatment, and response data were collected.

Results: Of 182 patients, 78% were p16-positive, were younger (predominantly male), mostly former or non-smokers, and had a more advanced nodal stage. With a median follow-up of 68 months, contralateral recurrence (CLR) rates were low (3.5% p16-positive versus 2.5% p16-negative, p=0.63). Overall survival (OS) was 74% for p16-positive versus 54% for p16-negative subjects (p=0.01), but all other outcomes were similar. Analysis amongst only p16-positive subjects revealed URT was delivered to 37%, with CLR rates of 7.5% versus 1.1% for those treated with BRT, p=0.05. Of the four p16-positive subjects treated with URT who developed contralateral recurrences, three were managed with neck dissection (two disease-free and one died of lung metastases) and one received palliative radiation to the neck and distant metastatic site. All disease control and survival outcomes were similar between those treated with URT versus BRT.

Conclusion: While CLRs remain rare overall, there appears to be a slightly increased rate among HPV-positive subjects treated with URT. However, overall outcomes do not appear to be impacted, suggesting that URT remains a reasonable approach in HPV-positive subjects.

## Introduction

There is known recognition of the role of human papillomavirus (HPV) in the etiology of head and neck cancers, notably those arising from the tonsil, base of tongue or elsewhere in the oropharynx [1]. Age-adjusted incidence of cancers of the oral cavity and larynx have declined along with tobacco usage. In contrast, oropharyngeal cancer incidence has increased, particularly in men, over a similar time period [2-3]. Increasing numbers of oropharyngeal cases are likely to be HPV-positive, although testing for HPV, or a surrogate, was not routinely conducted during the study period. Compared with HPV-negative oropharyngeal cancers, HPV-positive patients tend to be younger and use less alcohol and tobacco. HPV-positive cancers tend to present with a small, or occult, primary tumour, but frequently with large nodal involvement [4-5]. As a group, they also have a relatively favourable response to therapy with improved locoregional tumour control, improved disease-specific survival, and improved overall survival following radiotherapy (RT) or surgery when compared to HPV-negative cancers [6-9].

The use of unilateral RT (URT) techniques (also commonly known as ipsilateral RT) to treat patients with carcinoma of the tonsil reduces acute and late toxicity relative to bilateral techniques. In addition to producing less acute mucositis, URT reduces long-term damage to the salivary glands [10-12]. Sparing of one submandibular gland is beneficial, as these glands produce most of the resting saliva [13]. URT also reduces the dose to the contralateral carotid artery, potentially reducing the risk of stroke [14]. Finally, it has also been shown to reduce long-term dysphagia [12]. In the pre-HPV-era literature (namely, before the 2000s when smoking was the main driver of head and neck cancers), based on retrospective series comparisons, ipsilateral techniques gave results at least as good as those reported with bilateral techniques, with a low risk of failure in the contralateral neck [15-16]. There is no definitive evidence of increase in the risk of contralateral lymph node positivity in the HPV-era [17]; however, given the propensity of HPV-positive oropharyngeal cancer to spread at an early stage to the lymph nodes, there is a potential for increased risk associated with unilateral radiation. The purpose of this project is to explore the validity of URT in the HPV-era.

## Materials and methods

Records for all patients referred to the British Columbia Cancer Agency (BCCA) with squamous cell carcinoma (SCC) of the tonsil between January 1, 2001 and December 31, 2007 were retrieved from the provincial database. Search parameters were ICD codes C10.2 (lateral wall), C09.9 (tonsil), C09.0 (tonsillar fossa), and C09.1 (tonsillar pillars). Those who were of non-squamous histology, incorrectly classified with respect to tumour site, or not treated at BCCA were excluded. Electronic narrative patient charts were used to gather data on patient and treatment characteristics as well as outcomes. RT treatment plans were used to confirm treatment volumes where narrative charts were incomplete.

HPV status was determined through retrospective p16 immunohistochemistry (IHC) staining, given the recognised correlation between HPV DNA and p16 protein expression [5, 18]. Testing was carried out on available tissue samples, selecting those with the highest likelihood of having adequate tissue content. Four micrometre sections of formalin-fixed paraffin embedded tissues were analysed using a tissue microarray approach. p16^INK4a^ IHC was determined using mouse monoclonal primary antibodies from the CINtec(R) Cytology Kit (Roche mtm laboratories AG, Heidelberg, Germany). Those with IHC staining above 30% of cells were considered to be positive.

Staging investigations routinely consisted of flexible endoscopy, biopsy, CT scan of the neck, and chest x-ray. Examination under anesthesia (EUA) was only undertaken when a primary site was not easily indentified. After 2005, PET scans began to be used, but very infrequently. Less than 10% of patients in the study underwent either a diagnostic or routine follow-up PET scan.

Patients were CT simulated in the supine position with mask fixation. The usage of bite blocks was not uniform and varied by the regional centre. Gross tumour volumes were contoured using the CT simulation scan, and PET scan where available. A 5-7 mm margin was added for clinical target volumes and a 5-7 mm margin for planning target volumes. Subjects were treated with 66-70 Gy in 2 Gy daily fractions (46%), 60 Gy in 2.4 Gy daily fractions (42%), or more rarely, 55 Gy in 2.75 Gy daily fractions (7%) or 66 Gy in 2 Gy fractions concomitant boost (6%). These dose levels were to gross disease, with elective nodal volumes treated to 50-60 Gy. With respect to RT techniques, treatment was delivered with 3D conformal radiation therapy (3DCRT) or in the more modern era, intensity-modulated radiation therapy (IMRT). Dose and fractionation schedules, as well as the decision to use either ipsilateral or bilateral irradiation, was at the discretion of the treating oncologist. However, primary tumours with a significant extension on to the soft palate or base of tongue, or extending to within 1 cm of the midline were given bilateral treatment, as were those with bilateral lymph node disease. In unilateral treatments, the dose to the contralateral submandibular and parotid glands was < 20 Gy whether treated with 3DCRT or IMRT.

Concomitant chemotherapy regimens were either Cisplatin 100 mg/m^2^ on Days 1, 22, and 43, or Carboplatin 70 mg/m^2_,_^ along with 5-FU 600 mg/m^2^ on Days 1-4, 22-25, and 43-46. Carboplatin was used for subjects with renal impairment who could not receive Cisplatin.

All outcomes were measured from the date of diagnosis. Time to local, regional (ipsilateral or contralateral), or distant failure, death from disease or another cause, or last follow-up was recorded. Those with persistent disease treated with curative intent salvage treatment were not recorded as relapses unless they later had a separate failure. Those whose persistent disease was treated with palliative intent were defined as locoregional failures at the end of the treatment date. Disease-free survival (DFS) was defined as time to local, regional, or distant failure while disease-specific survival (DSS) was defined as time to death from tonsil SCC. Outcomes were censored at the date of the last contact.

All analysis was completed using Statistical Package for Social Sciences version 14.0. In addition to descriptive analysis, independent sample t-tests and Pearson Chi-square cross tabulations were used to make comparisons between both p16-positive and negative subjects, as well as those treated with unilateral versus bilateral RT. Actuarial outcomes analysis was completed using Kaplan-Meier and log-rank analysis.

This study was approved by the British Columbia Cancer Agency Institutional Research Ethics Board (approval #H11-00364). Informed patient consent had been obtained at the time of treatment.

## Results

A total of 405 cases of tonsil cancer between 2001 and 2007 were retrieved from the provincial database. Eighty-three patients were excluded due to classification errors or not receiving treatment at the agency. One hundred and forty patients were excluded because p16 status could not be determined – either due to an inability to procure the pathology sample or insufficient tissue to allow testing. A total of 182 charts were then reviewed.

### p16-positive versus p16-negative

Of these, 142 patients (78%) were p16-positive. These subjects tended to be younger, male, former or non-smokers, and had more advanced nodal and overall stage. RT, chemotherapy, and surgery treatment characteristics were similar (Table 1). Rates of contralateral recurrence (CLR) were low overall and did not differ by p16 status (3.5% p16-positive versus 2.5% p16-negative, p = 0.75). With a median follow-up of 68 months for all patients (73.5 months for p16-positive and 53.5 months for p16-negative), local control (LC), locoregional control (LRC), DFS, and DSS were statistically similar, but the five-year overall survival (OS) was 74% for p16-positive versus 54% for p16-negative subjects (p=0.01) (Table 2).

**Table 1:** Baseline patient and treatment characteristics, by p16 status CRT = Chemoradiation, RT = Radiation, UND = unilateral neck dissection

Characteristic	p16-positive (n=142)	p16-negative (n= 40)	p-value
Mean age at diagnosis	55.7	59.5	0.02
Gender			<0.01
Male	117 (82%)	23 (58%)	
Female	25 (18%)	17 (43%)	
T Stage			0.37
T1	44 (31%)	8 (20%)	
T2	67 (47%)	19 (48%)	
T3	24 (17%)	11 (28%)	
T4	7 (5%)	2 (5%)	
N Stage			< 0.01
N0	26 (18%)	18 (45%)	
N1	26 (18%)	5 (13%)	
N2	75 (53%)	15 (38%)	
N3	15 (11%)	2 (5%)	
Stage			0.03
I	3 (2%)	4 (10%)	
II	18 (13%)	9 (23%)	
III	28 (20%)	10 (25%)	
IVA	78 (55%)	15 (38%)	
IVB	15 (11%)	2 (5%)	
Smoking			< 0.01
Current/quit less than 1 year	42 (30%)	31 (78%)	
Former (quit more than 1 year)	55 (39%)	2 (5%)	
Never	45 (32%)	7 (18%)	
Radiation			
< 60 Gy	66 (46%)	21 (53%)	0.5
61-70 Gy	76 (54%)	19 (48%)	
Unilateral	53 (37%)	17 (43%)	0.56
Bilateral	89 (63%)	23 (58%)	
RT alone	83 (59%)	29 (73%)	0.11
CRT	59 (42%)	11 (28%)	
Chemotherapy			0.61
Cisplatin-based	47 (80%)	8 (73%)	
Carboplatin-based	12 (20%)	2 (27%)	
Surgery			0.51
None	128 (90%)	39 (98%)	
Primary resection	1 (< 1%)	0	
Post-RT primary and UND	1 (<1%)	0	
Post-RT UND	12 (9%)	1 (3%)	

**Table 2:** Five year outcomes by p16 status and laterality of radiation

	p16 status			Laterality of Radiation		
	p16-positive	p16-negative	Log rank p-value	URT	BRT	Log rank p-value
LC	90%	83%	0.24	89%	87%	0.69
LRC	84%	79%	0.34	83%	83%	0.94
DFS	78%	74%	0.43	78%	78%	0.99
DSS	82%	76%	0.36	83%	79%	0.26
OS	74%	54%	0.01	72%	68%	0.34

### Unilateral versus bilateral radiation

**Figure 1:**
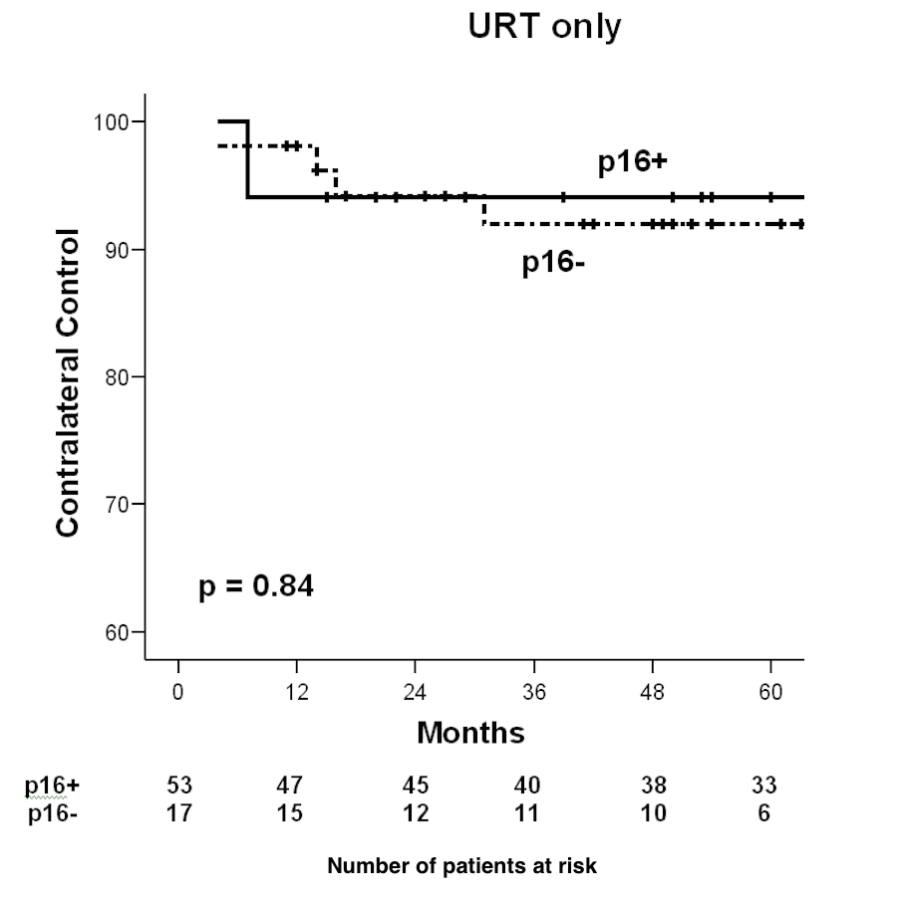
Contralateral recurrences for URT subjects, by p16 status

Overall, 70 subjects (38%) were treated with URT. URT treated patients were similar in gender and age, but of earlier T, N, and overall stage, as compared to those who received BRT. In addition, the total dose of RT was more likely to be less than or equal to 60 Gy in those receiving URT. While those who received URT were more likely to have CLR (7.1%) than those treated with bilateral RT (BRT) (0.9%) (p = 0.02), the LC, LRC, DFS, DSS, and OS were not worse for URT subjects (Table 2). Amongst URT subjects only, both crude CLR (7.5% p16-positive and 5.9% p16-negative, p=0.82) and actuarial CLR did not differ by p16 status (Figure 1).

### p16-positive only, unilateral vs. bilateral radiation

Among the 142 p16-positive subjects, 53 were treated with unilateral RT (URT). Those treated with URT versus BRT were similar in age and gender, but those treated with URT were of less advanced nodal and overall stage. Compared with those who received BRT, URT subjects were again more likely to have been treated with RT doses less than or equal to 60 Gy. Those treated with URT were more likely to be treated with RT alone, but the choice of the chemotherapeutic regimen was similar (Table 3).

**Table 3:** Baseline characteristics of p16-positive subjects, by laterality of radiation

Characteristic	Unilateral RT (n=53)	Bilateral RT (n=89)	p-value
Mean age at diagnosis	55.2	56.0	0.65
Gender			0.09
Male	43 (81%)	74 (83%)	
Female	10 (19%)	15 (17%)	
T Stage			< 0.01
T1	22 (42%)	22 (25%)	
T2	28 (53%)	39 (44%)	
T3	3 (6%)	21 (24%)	
T4	0	8 (8%)	
N Stage			< 0.01
N0	15 (28%)	11 (12%)	
N1	18 (34%)	8 (9%)	
N2a	7 (13%)	9 (10%)	
N2b	11 (21%)	36 (40%)	
N2c	0	12 (14%)	
N3	2 (4%)	13 (15%)	
Stage			< 0.01
I	1 (2%)	2 (2%)	
II	13 (25%)	5 (6%)	
III	19 (36%)	9 (10%)	
IVA	18 (34%)	60 (67%)	
IVB	2 (4%)	13 (15%)	
Smoking			0.03
Current/quit less than 1 year	13 (25%)	29 (33%)	
Former (quit more than 1 year)	28 (53%)	27 (30%)	
Never	12 (23%)	33 (37%)	
Radiation			
< 60 Gy	35 (66%)	31 (35%)	< 0.01
61-70 Gy	18 (34%)	58 (65%)	
RT alone	42 (79%)	41 (46%)	<0.01
CRT	11 (21%)	48 (54%)	
Chemotherapy			0.30
Cisplatin-based	19 (91%)	37 (77%)	
Carboplatin-based	1 (9%)	11 (23%)	
Surgery			0.29
None	51 (96%)	77 (87%)	
Primary resection	0	1 (1%)	
Post-RT primary and UND	0	1 (1%)	
Post-RT UND	2 (4%)	1 0 (11%)	

After completion of primary treatment, 81% of p16-positive subjects had a complete clinical response at first assessment after completion of primary treatment. There were more complete responses in the URT group (93% vs. 74%, p < 0.01).            

**Figure 2:**
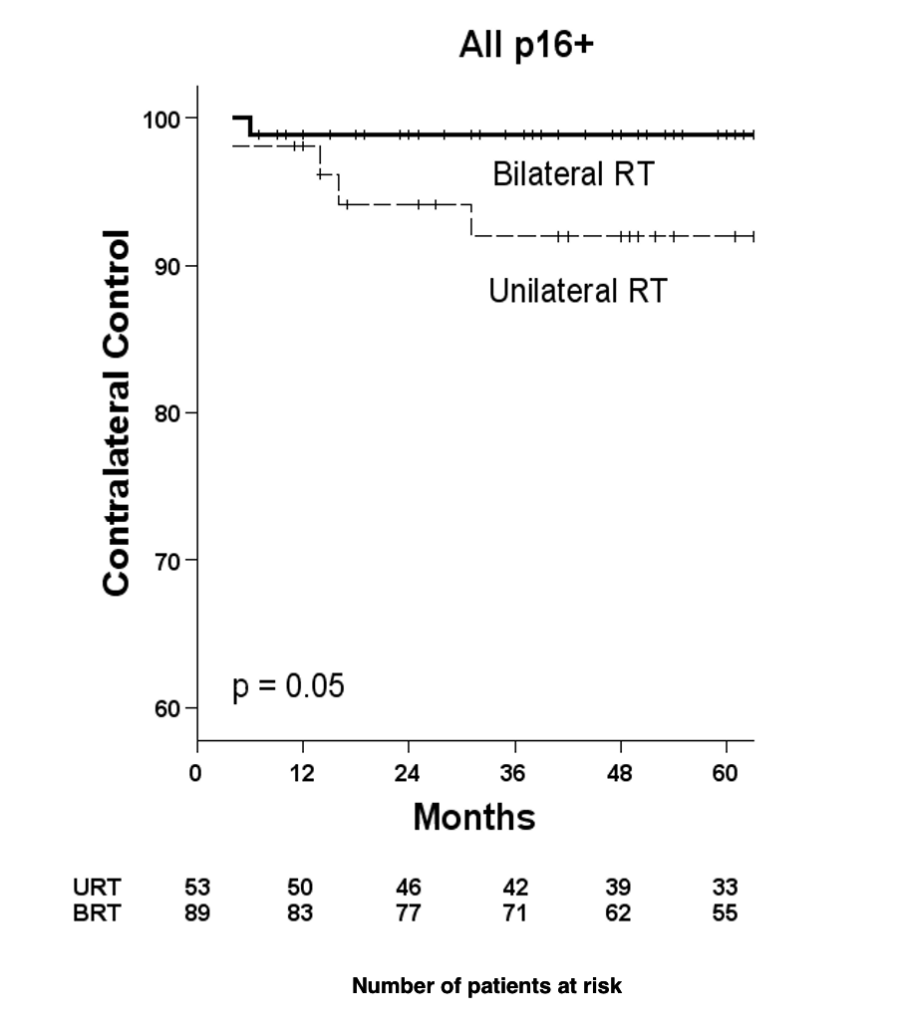
Contralateral recurrences for p16-positive subjects, by laterality of RT

CLRs were seen in four (7.5%) subjects treated with URT versus one (1.1%) treated with BRT (Figure 2). Three of the four URT subjects with CLR were treated with a neck dissection while one received palliative RT for the CLR and concurrent pulmonary metastases (and died of his disease over one year later). Of the three patients who underwent neck dissection, one was disease-free at last contact, one died of pulmonary metastases, and one died of a second primary cancer of the lung. The subject treated with BRT underwent bilateral neck dissection but developed ipsilateral subcutaneous metastases within five months and died of their disease.

Among the p16-positive subjects treated with URT, CLR did not correlate with tumour, nodal, or overall stage. Certain subgroups had very small numbers limiting this analysis (Table 4).

**Table 4:** Contralateral recurrences by T, N, and overall stage for p16-positive subjects treated with unilateral radiation therapy

	n	CLR (%)	p-value
T1	22	1 (5%)	0.10
T2	28	2 (7%)	
T3	3	1 (33%)	
N0	15	0	0.26
N1	18	3 (17%)	
N2a	7	1 (14%)	
N2b	11	0	
N2c	0	0	
N3	2	0	
Stage I	1	0	0.45
Stage II	13	0	
Stage III	19	3 (16%)	
Stage IVA	18	1 (6%)	
Stage IVB	2	0	

**Figure 3:**
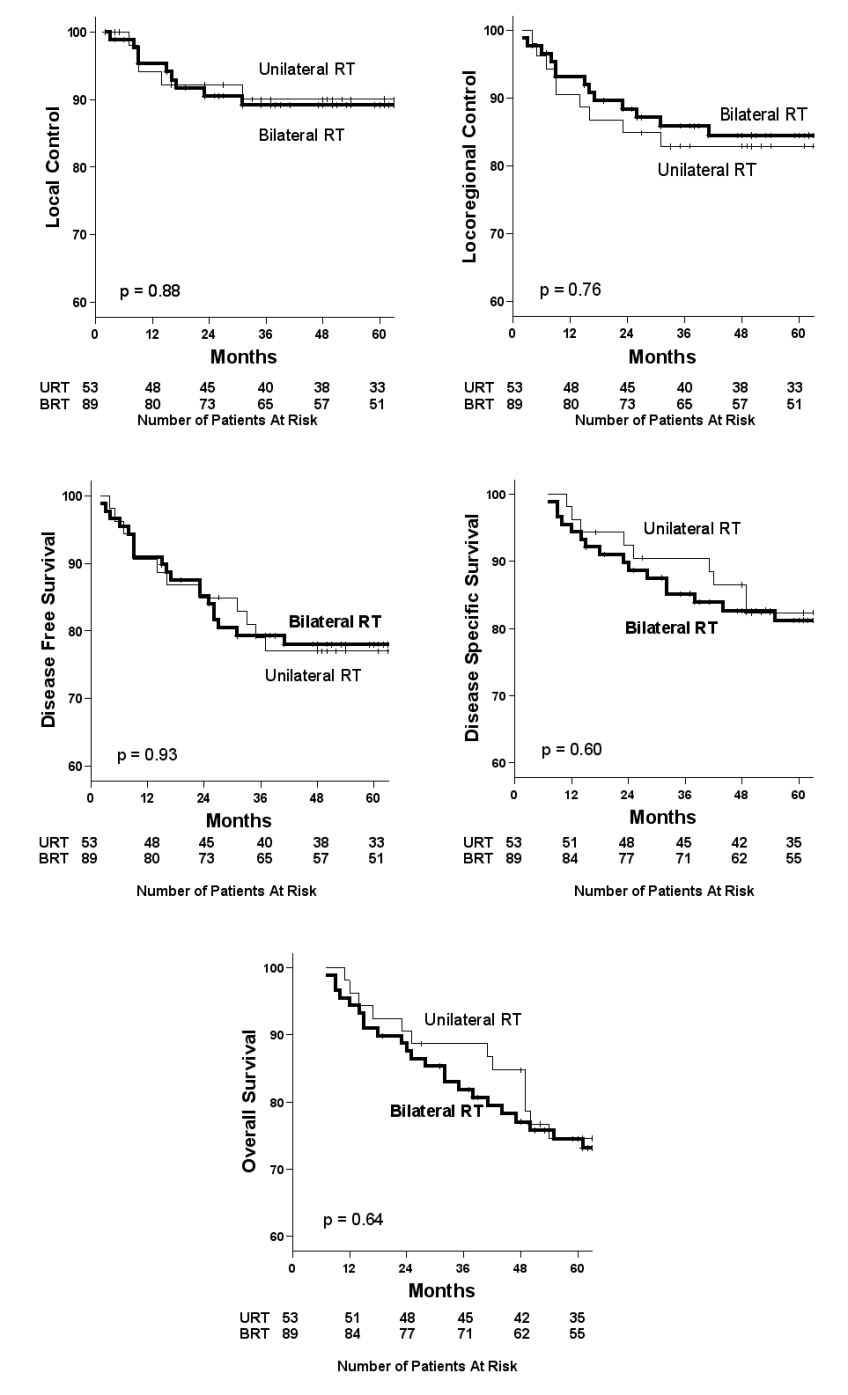
Outcomes for p16-positive subjects, by laterality of RT

LC, LRC, DSS, DFS, and OS curves are shown in Figure 3. Despite the difference in contralateral recurrences, no differences in overall outcomes were seen for those treated with URT versus BRT.

## Discussion

We, and others, have previously published evidence that supports the practice of ipsilateral radiation therapy for selected cases of tonsillar SCC [15-16, 19-21]. In a broader context, there is also evidence to support this practice in patients treated with primary surgery, and not just amongst tonsillar primaries [22]. Although there is a variability in the stage of cancers included, the radiotherapy series all report rates of CLR less than 5%. We utilised similar criteria for the selection of URT but saw a higher proportion of more advanced nodal disease in our population. However, overall CLR rates in this series remain comparable, at 7.1% for URT and 0.9% for BRT. While these results are statistically different, they remain low overall, and less than 10% (which has previously been accepted as a reasonable threshold for elective neck irradiation) [23].

In the modern era with increasing use of altered fractionation, concurrent chemotherapy, and increasingly conformal radiotherapy techniques, outcomes have improved from five-year DSS of approximately 60% in the earlier series to 75-80% in the more recent publications.

With increasing rates of HPV-associated cancer and the accompanying presentation of more advanced nodal disease [4-5], the question is whether we are treating a new disease which would require a shift in the treatment approach, namely, routine bilateral neck irradiation. In this series, amongst the p16-positive subjects, the risk of developing contralateral recurrence remained low overall (3.5%) and was very similar to the rates seen in p16-negative subjects (2.5%). While URT was associated with higher rates of CLR in p16-positive subjects (7.5%) than with BRT (1.1%), long-term outcomes were not affected, as LC, LRC, DFS, DSS, and OS remained similar between the two radiation techniques. This is likely due to both overall low rates of CLR and the efficacy of salvage treatment. Three of the four contralateral recurrences that developed in patients after unilateral radiation were effectively treated with a neck dissection, which provided control of the neck disease.

If we are to accept a slightly higher rate of CLR at the cost of a higher rate of salvage neck dissection, it is important to note the morbidity associated with this combination of treatment modalities. The late effects associated with neck radiation have been well-described to include xerostomia – which causes an increased risk of dental caries, neck fibrosis, dysphagia, and cerebrovascular events [10-12, 14, 24]. With salvage surgery, risks such as delayed healing, wound infection, or major bleeding must be considered. In general, these risks are estimated to be two to three times higher post-irradiation [24]. The most important long-term morbidity after neck dissection is shoulder dysfunction. This usually takes the form of shoulder pain and impairments in strength and range of motion [25]. While there is a lack of comparative evidence, it is felt that neck dissections for contralateral nodal recurrences can be done with little morbidity [26].

The American College of Radiology has published guidelines on how to select patients with tonsil SCC for URT. In it, nodal stage greater or equal to N2b is considered criteria for BRT [27]. However, in our data when examining the outcomes by tumour and nodal stage, it is reassuring that there is no significant difference in CLR rates. While more advanced T stage trended towards increased CLR rates, this was not true for advanced N stage. In fact, in our series, none of the p16-positive subjects with N2b who were treated with URT developed contralateral recurrences. In a small series from Delaware, patients with node-positive tonsil cancer, up to and including N2b disease, were treated with URT. Many (70%) were treated after surgery, including neck dissection. No contralateral nodal failures were observed, leading the authors to conclude that the extent of the primary tumour is a more important predictor of failure than the nodal status [28]. Two other series lend support to our findings. Twenty patients from a Korean series treated unilaterally had T1-T3, N0-N2a disease. The primary tumours did not cross the midline and had less than 1 cm of tumour invasion into the soft palate or base of tongue (BOT). With a median follow-up of 64 months, the five-year local progression-free survival, distant metastasis-free survival, and progression-free survival rates were 95%, 100%, and 95%, respectively. The exclusion of extensive palate or BOT invasion was felt to be a possible reason for the excellent disease outcomes [29]. Furthermore, commentary and data from Peter MacCallum Cancer Centre details subjects with T1-4, N0-2b or N3 were treated with URT and none of the patients experienced contralateral nodal failure [21, 30].

In the above series, PET staging was widely used. In our older cohort, only twelve (6.5%) subjects underwent staging PET evaluation prior to treatment, leading to a plausible risk of nodal under-staging. In today’s era of routine PET imaging and, therefore, increased staging accuracy, the possibility of delivering radiotherapy to a patient who harbours contralateral nodal disease is presumably lower. In fact, El Khodary, et al, have shown that PET-CT yields additional diagnostic information in just under half of HNSCC patients and can modify the radiotherapy plan in up to a quarter [31].

To the best of our knowledge, this is the first series to review outcomes of URT by HPV status. A recent SEER analysis attempted to address this question by examining the trends associated with tumour size and nodal presentation [17]. The data demonstrated an increase in advanced regional disease in all tumour stages, but not necessarily an increase in bilateral nodal disease. Those authors concluded that there is no justification for altering the current practice of URT for well-lateralised disease. The results of our series support their conclusion.

While intrinsic differences between HPV-positive and HPV-negative head and neck squamous cell carcinoma (HNSCC) is not the focus of this paper, it is of interest that, despite similar rates of disease control, p16-positive subjects were shown to have superior OS. This is likely related to the differences in patient characteristics between the two groups, with p16-positive subjects tending to be younger (fewer comorbidities) and less likely to be smokers (lower risk of other malignancies).

## Conclusions

Our findings confirm the relatively good prognosis associated with p16-positive squamous cell carcinomas of the tonsil compared to malignancies that are p16-negative. Overall, the rate of contralateral recurrence following unilateral radiation therapy was low, and there was no difference in the rates of disease control and overall survival when compared to patients who received bilateral treatment. Furthermore, even when considering only p16-positive subjects, unilateral versus bilateral radiation therapy did not alter disease-free or overall survival outcomes. While advanced T stage appeared to trend with increased rates of contralateral recurrence, no such association was seen for advanced nodal disease. Despite the increased burden of nodal disease associated with p16 positivity, we believe that unilateral radiation therapy in well-lateralised squamous cell carcinoma of the tonsil remains a safe and effective treatment. These conclusions are within the context of a retrospective review with small numbers of subjects with very advanced nodal disease.
